# Soluble low-density lipoprotein receptor-related protein 1 as a surrogate marker of carotid plaque inflammation assessed by ^18^F-FDG PET in patients with a recent ischemic stroke

**DOI:** 10.1186/s12967-022-03867-w

**Published:** 2023-02-19

**Authors:** Eduardo Garcia, Pol Camps-Renom, Núria Puig, Alejandro Fernández-Leon, Ana Aguilera-Simón, Aleyda Benitez-Amaro, Arnau Solé, David Vilades, José Luis Sanchez-Quesada, Joan Martí-Fàbregas, Elena Jiménez-Xarrié, Sonia Benitez, Vicenta Llorente-Cortés

**Affiliations:** 1 Lipids and Cardiovascular Pathology, Institut d’Investigacions Biomèdiques de Barcelona (IIBB)-Spanish National Research Council (CSIC), Institut d’Investigació Biomèdica Sant Pau (IIB SANT PAU), Sant Quintí 77-79, 08041 Barcelona, Spain; 2 Universitat Autònoma de Barcelona, Barcelona, Spain; 3 Stroke Unit, Department of Neurology, Hospital de la Santa Creu i Sant Pau, IIB SANT PAU, Barcelona, Spain; 4 Cardiovascular Biochemistry, Institut d’Investigació Biomèdica Sant Pau (IIB SANT PAU), Sant Quintí 77-79, 08041 Barcelona, Spain; 5 Department of Biochemistry and Molecular Biology, Faculty of Medicine, Universitat Autònoma de Barcelona (UAB), Building M, Cerdanyola del Vallés, Barcelona Spain; 6 Department of Nuclear Medicine, Hospital de la Santa Creu i Sant Pau, IIB SANT PAU, Barcelona, Spain; 7 Cardiac Imaging Unit, Department of Cardiology, Hospital de la Santa Creu i Sant Pau, IIB SANT PAU, Barcelona, Spain; 8 CIBER of Cardiovascular (CIBERCV), Instituto de Salud Carlos III, Madrid, Spain; 9 CIBER of Diabetes and Metabolic Diseases (CIBERDEM), Instituto de Salud Carlos III, Madrid, Spain

**Keywords:** Soluble LRP1, ^18^F-FDG PET, Carotid atherosclerosis, Ischemic stroke recurrence, Vascular inflammation

## Abstract

**Background:**

^18^F-fluorodeoxyglucose positron emission tomography (^18^F-FDG PET) identifies carotid plaque inflammation and predicts stroke recurrence.

**Aim:**

Our aim was to evaluate the performance of soluble low-density lipoprotein receptor-related protein 1 (sLRP1) as an indicator of carotid plaque inflammation.

**Methods:**

A prospective study was conducted among adult patients with recent (< 7 days) anterior circulation ischemic stroke and at least one atherosclerotic plaque in the ipsilateral internal carotid artery. Patients underwent an early (< 15 days from inclusion) ^18^F-FDG PET, and the maximum standardized uptake value (SUVmax) within the plaque was measured. sLRP1 levels were measured in plasma samples by ELISA. The association of sLRP1 with SUVmax was assessed using bivariate and multivariable linear regression analyses. Hazard ratios (HR) were estimated with Cox regression to evaluate the association between circulating sLRP1 and stroke recurrence.

**Results:**

The study was conducted with 64 participants, of which 57.8% had ≥ 50% carotid stenosis. The multivariable linear and logistic regression analyses showed that sLRP1 was independently associated with (i) SUVmax within the plaque (β = 0.159, 95% CI 0.062–0.257, *p* = 0.002) and (ii) a probability of presenting SUVmax ≥ 2.85 g/mL (OR = 1.31, 95% CI 1.00–1.01, *p* = 0.046), respectively. Participants with stroke recurrence showed higher sLRP1 levels at baseline [6447 ng/mL (4897–11163) vs. 3713 ng/mL (2793–4730); *p* = 0.018].

**Conclusions:**

sLRP1 was independently associated with carotid plaque inflammation as measured by ^18^F-FDG PET in patients with recent ischemic stroke and carotid atherosclerosis.

**Supplementary Information:**

The online version contains supplementary material available at 10.1186/s12967-022-03867-w.

## Introduction

Cardiovascular disease is a main cause of death and disability worldwide [1]. Atherosclerosis from the internal carotid artery is responsible for 15–20% of all ischemic strokes [2]. Patients with recent symptomatic carotid plaques show a three-fold risk of early stroke recurrence as compared to other stroke subtypes [3]. In addition, stroke survivors are at risk of coronary events or recurrent strokes despite guideline-based treatments [4–6]. Therefore, there is a need for new diagnostic biomarkers to help assess the residual risk prediction of vascular events after an atherothrombotic stroke.

Inflammation has emerged as a key feature of carotid plaque vulnerability. Of note, it can be measured non-invasively by ^18^F-fluorodeoxyglucose positron emission tomography (^18^F-FDG PET) [7]. A recent study revealed ^18^F-FDG PET to be a mainstay technique for stroke prognosis, based on an independent association between maximal standardized uptake value (SUVmax) within the plaque and early stroke recurrence in patients with carotid stenosis [8, 9]. Interestingly, a SUVmax threshold of 2.85 g/mL discriminates stroke recurrence with high sensitivity, even in patients with moderate stenosis. However, some intrinsic limitations of this imaging technique, such as its high cost and limited availability, may hinder its implementation in clinical practice.

In this regard, finding plasma biomarkers associated with plaque inflammation assessed by ^18^F-FDG PET could be of great interest, as they would eventually serve as screening tools as well as markers for monitoring response to preventive treatments. Recent reports show that circulating concentrations of fractalkine (FKN), soluble vascular cell adhesion molecule 1 (sVCAM-1), and soluble intercellular cell adhesion molecule 1 (sICAM-1) are associated positively with higher degrees of plaque inflammation; in particular, sICAM-1 predicts with high sensitivity the risk of finding highly inflamed carotid plaques and the risk of stroke recurrence within one year [10].

Soluble low-density lipoprotein receptor-related protein 1 (sLRP1) has been recently postulated to be a predictive biomarker for coronary artery disease (CAD) risk in a case-cohort study based on the follow-up of the REGICOR population-based cohort, in which no participants had a previous history of cardiovascular disease [11]. sLRP1 is generated by constitutive or induced intramembrane proteolysis of the cell receptor [12] mediated by several metalloproteinases [13, 14]. LRP1 shedding can be induced by atherogenic lipoproteins in human coronary vascular smooth muscle cells [15], by tissue plasminogen activator (tPA) in astrocytes [16], and by inflammatory mediators in macrophages [17]. In the context of stroke, sLRP1 seem to play a neuroprotective role in hemorrhagic stroke by removing heme-hemopexin complexes, but it may also induce deleterious effects in ischemia by eliciting blood–brain barrier permeability and glial inflammation [18].

The soluble LRP1 form seems to have functions that are independent of those of the cellular LRP1 form [18]. The cellular form is involved in the regulation of vascular integrity [18, 19] and vascular lipoprotein metabolism [20–22]. In particular, cellular LRP1 receptor plays a crucial role in the generation of foam cells, which become proinflammatory cells [22, 23] with high capacity to release sLRP1 [15, 17, 22]. The role of sLRP1 is rather unknown and could be, at least partially determined by the competition with membrane LRP1 for certain ligands.

On the basis of these previous results, the aims of this work were to determine: (i) the performance of sLRP1 as an indicator of carotid plaque inflammation evaluated by ^18^F-FDG PET and (ii) the value of sLRP1 as a prognostic biomarker for stroke recurrence.

## Methods

### Study participants and study design

An observational cohort study (NCT03218527) was conducted with consecutive adult patients with a recent anterior circulation ischemic stroke and carotid atherosclerosis. The study was conducted in our center between January 2016 and March 2019. It was approved by the Ethics Committee of Hospital Santa Creu i Sant Pau (IIBSP-LRB-2017-54), and patients gave written consent for participation. The study was performed in accordance with the Helsinki Declaration.

Participants of the current study were the same who had been included in a previous publication [10]. Briefly, patients were required to fulfill the following inclusion criteria: (1) the age limit was fixed in ≥ 50 years to minimize the inclusion of non-atherosclerotic vasculopathies; (2) anterior circulation ischemic stroke or transient ischemic attack (TIA) within the seven days preceding the study inclusion; (3) at least one atherosclerotic plaque in the internal carotid artery (ICA) on the side consistent with stroke symptoms, regardless of the degree of stenosis [24]; and (4) a modified Rankin Scale (mRS) score < 4 before inclusion. Exclusion criteria were: (1) the presence of definitive cardioembolic, lacunar, or unusual stroke etiology according to the TOAST criteria [25]; (2) hemodynamic stroke/TIA; (3) prior carotid surgery or stenting; (4) life-expectancy < 1 year; or (5) suspicion of concomitant infections at the time of blood extraction.

The following variables related to vascular risk and stroke characteristics were recorded for all participants: (1) age and sex; (2) medical history, including hypertension, diabetes, dyslipidemia, prior stroke/TIA, coronary artery disease, smoking, and alcohol abuse; (3) previous treatments; (4) National Institutes of Health Stroke Scale (NIHSS) score at admission; (5) body mass index (BMI); (6) adherence to regular physical exercise, according to the physician-based assessment and counseling for exercise (PACE) scale [26]; (7) Mediterranean diet adherence according to the PREDIMED score [27]; (8) mRS score at inclusion; (9) stroke etiology according to the TOAST criteria after a diagnostic work-up that included a 24-h (or longer) electrocardiogram monitoring, an echocardiogram, and an ultrasound carotid examination; and (10) results from the admission blood test, including renal function, blood cell counts, hemostasis, and lipid profile.

### Atherosclerosis imaging by ^18^F-FDG PET and assessment of plaque inflammation in the internal carotid artery

All stroke patients underwent a baseline medical assessment by trained study personnel and a carotid ^18^F-FDG PET/CT study in less than 15 days from the inclusion. The blood sample was collected at day 7 ± 1 from the index stroke. The treating clinicians provided medical and revascularization treatments according to current guidelines [28].

Carotid ^18^F-FDG PET was performed in a Philips Gemini TF TOF 64 PET/CT (Philips Medical System, Eindhoven, and Netherlands) as previously described [8, 9]. The examinations were performed after a fast of at least six hours. PET scans were not performed if pre-PET blood glucose levels exceeded 10 mmol/L. Two hours before image acquisition, 320 MBq of ^18^F-FDG was administered. The uptake phase was standardized with the patient resting. PET images were acquired in 3-dimensional mode in 2-bed positions for 10 min each. CT angiography was usually done at admission, sometimes before PET/CT. CT angiography and PET images were co-registered afterwards to assess the slice of maximal plaque stenosis.

^18^F-FDG activity was measured in 10 regions of interest, which were defined relative to the slice of maximal stenosis on the co-registered CT angiography, corresponding to a 1 mm axial plaque slice (5 distal and 5 proximal). ^18^F-FDG was quantified using standardized uptake values [SUV g/mL, defined as measured uptake (MBq/mL)/injected dose (MBq) per patient weight (g)]. The single hottest slice was defined as the axial slice with maximal SUV uptake (SUVmax) [7].

### Plasma and serum determinations

Peripheral blood samples from the participants were collected at day 7 ± 1 after the stroke/TIA. Plasma was collected in ethylenediaminetetraacetic acid (EDTA)-containing Vacutainers and serum was collected in Serum Separator Tubes coated with clot activator. Tubes were centrifuged at 1500*g* for 15 min at 4 ℃, and aliquots were frozen at – 80 ℃ until analysis.

Serum lipid profile (triglycerides, total cholesterol, low-density lipoprotein cholesterol (LDLc), and high-density lipoprotein cholesterol (HDLc), creatinine, hemoglobin, and high sensitivity CRP (hsCRP) were measured in an autoanalyzer (Alinity ci-series, Abbott Core Laboratory, Chicago, Illinois, USA). Glycated hemoglobin (Hba1c) was quantified using high-performance liquid chromatography in total blood.

### Measurement sLRP1 concentrations

sLRP1 concentrations were measured in frozen plasma using commercially available enzyme-linked immunosorbent assay (ELISA) (Uscn Life Science Inc. China) according to the manufacturer’s recommendations. The assay had a within- and between-assay coefficient of variation lower than 10%, and 12%, respectively, and a limit of detection of 0.156 ng/mL. The assay showed no significant cross-reactivity or interference between LRP1 and analogues.

### Follow-up and outcomes

Standardized follow-up assessments were performed for all participants on day 90 from the index stroke, and every 6 months thereafter until the study terminated.

The primary outcome was to determine the association between clinical, imaging, and laboratory variables (including sLRP1 levels) collected in the study and carotid plaque inflammation assessed by ^18^F-FDG PET/CT, as previously described.

The secondary outcome was any anterior circulation ipsilateral recurrent stroke along patient follow-up. A recurrent stroke was defined as a new sudden onset of persistent (≥ 24 h) neurologic deficit after the index event, or a new persistent or transient neurologic deficit with imaging confirmation of a new cerebral infarction. Only strokes unrelated to a revascularization procedure (defined as any event > 24 h after carotid revascularization) were considered outcomes. During the follow-up, nine stroke recurrences were registered in our population that represent a risk of 14.1%. Of them, five recurrences occurred before PET (7.8%) and four recurrences occurred after PET (6.3%).

### Statistical analyses

Data were presented as mean and standard deviation (SD) for continuous variables, median and interquartile range for continuous variables not normally distributed, and frequencies (percentages) for categorical variables. Data normality was analyzed using the Kolmogorov–Smirnov test. Continuous variables were compared between groups using the Student’s *t*-test or the Wilcoxon Rank Sum test (when a non-parametric test was required). Categorical variable comparisons were performed using the chi-square test. The Spearman’s test was performed to evaluate the correlations between sLRP1 and other variables. The association between circulating sLRP1 levels and plaque inflammation assessed by SUVmax in the ^18^F-FDG PET/CT was analyzed. After a bivariate analysis including sLRP1 levels and all the variables collected in the study, we performed a multivariable analysis using linear regression. From a maximal model including all the variables showing a trend towards significance in the bivariate analysis (*p* < 0.1), a backward stepwise selection modeling was performed, and only variables with a significance level of *p* < 0.05 were kept in each iteration. Data were expressed as beta coefficient with its 95% CI and standardized beta (β). The following variables were log-transformed to achieve a normal distribution when constructing the linear regression models: SUVmax, sLRP1, triglycerides, LDLc, HDLc, and high sensitivity CRP.

The association of circulating sLRP1 and the probability of presenting an intraplaque SUVmax ≥ 2.85 g/mL was also studied. Bivariate and multivariable logistic regression analyses were performed using the same variables selected in the multivariable linear regression analysis. The results are presented as odds ratio (OR) and 95% confidence intervals (CI). ROC analyses were performed to compare the predictive value of sLRP1 and high-sensitivity C-reactive protein (hsCRP) to identify highly inflamed plaques.

Finally, a survival analysis was conducted using Cox regression to test the association between sLRP1 levels and stroke recurrence during the follow-up.

The statistical software package SPSS 15.0 for Windows (SPSS Inc., Chicago, IL, USA) and Stata v.15 (Texas, US) were used for the statistical analyses. Differences were considered statistically significant when *p* < 0.05.

## Results

### Clinical characteristics of the study population and sLRP1 levels

Clinical and biochemical parameters for the studied population are summarized in Additional file 1: Table S1. The study included 64 patients, 37 (57.8%) with a ≥ 50% carotid stenosis, and the remainder with mild stenosis (< 50%). Within the participant cohort, the average age was 74.9 ± 9.6 years, 75% were men, 82% had hypertension, 42% had type 2 diabetes mellitus, 59.3% had obesity, and 67.2% were current smokers.

The SUVmax was significantly higher in patients with more than 50% stenosis [2.74 g/mL (2.41–3.23) vs. 2.39 g/mL (2.23–2.69); *p* = 0.034], whereas there were no significant differences in the rest of clinical variables dichotomizing the participants according to stenosis degree (e.g., higher or lower than 50%). We found that sLRP1 levels were not different according to the degree of stenosis [stenosis ≥ 50%: 3915 ng/mL (3190–4730) vs. stenosis < 50%: 3596.7 ng/mL (2665–5130); *p* = 0.688].

Spearman’s test showed that sLRP1 positively correlated with triglycerides (r^2^ = 0.426, *p* < 0.001) and abdominal perimeter (r^2^ = 0.304, *p* < 0.024). In addition, the correlation of sLRP1 with some inflammatory molecules including sICAM1, sVCAM1 and FKN (found associated with plaque inflammation in the same cohort of patients) [9] was analyzed. In particular, sLRP1 positively correlated with sICAM-1 (*p* = 0.009) and showed a strong trend towards significance with sVCAM-1 (*p* = 0.087) and FKN (*p* = 0.066) (Table 1).

**Table 1:** Analysis of sLRP1 association with inflammatory and metabolic variables

	Correlation coefficient	p
sICAM-1	0.337	0.009
sVCAM-1	0.225	0.087
FKN	0.239	0.066
TG	0.426	< 0.001
Abdominal perimeter	0.304	< 0.024

The correlation coefficient and p-value were obtained by Spearman correlation test

*sLRP1* soluble low-density lipoprotein receptor-related protein 1, *sICAM-1* soluble intercellular adhesion molecule-1, *sVCAM-1* soluble vascular adhesion molecule-1, *FKN* fractalkine, *TG* triglycerides

### Circulating sLRP1 concentrations are associated with carotid plaque inflammation

Carotid plaque inflammation was assessed by ^18^F-FDG PET imaging in accordance with the study protocol for 53 of 64 participants (24 in the < 50% stenosis group, and 29 in the ≥ 50% stenosis group). In three cases, although ^18^F-FDG PET imaging was performed, the carotid inflammation was not assessable due to acquisition problems. In eight cases, ^18^F-FDG PET was not performed. In four cases, the patients underwent carotid revascularization before PET examination, and the other four patients refused to undergo PET.

We found a positive association between SUVmax and the following clinical variables: BMI (β = 0.008, 95% CI 0.001–0.015, *p* < 0.016), waist circumference (β = 0.003, 95% CI 0.001–0.005, *p* = 0.004), and PACE (β = − 0.015, 95% CI − 0.028–0.002, *p* = 0.023) (Additional file 1: Table S2), according to our previous study [9].

The strongest association was observed for sLRP1, which was also significantly and positively associated with carotid plaque inflammation (β = 0.192, 95% CI 0.094–0.290, *p* < 0.001) (Additional file 1: Table S2).

There were not differences in sLRP1 levels between patients with (n = 35) or without (n = 29) previous treatment with statins (p = 0.364). Spearman correlation analysis indicated that sLRP1 correlated with SUVmax only in the group previously treated with statins (coefficient correlation = 0.563; p = 0.001).

**Fig. 1:**
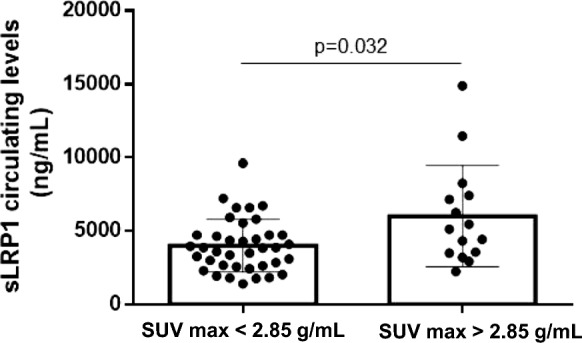
Circulating sLRP1 levels in patients with low (SUVmax < 2.85 g/mL) or high (SUVmax > 2.85 g/mL) carotid plaque inflammation. Patients were dichotomized according to SUVmax values by the cut-off point of 2.85 g/mL, and sLRP1 levels were measured in plasma-EDTA blood samples by ELISA. Results are shown as mean ± SD. sLRP1, soluble low-density lipoprotein receptor-related protein 1

The ROC sensitivity/specificity analysis from our previous study, [7] led a balanced cut-off point for predicting stroke recurrence of 2.85 g/mL. The dichotomization of SUVmax value by the cut-off point of 2.85 g/mL showed increased sLRP1 levels in patients with SUVmax ≥ 2.85 g/ml as compared to those with SUVmax < 2.85 g/mL [5130 ng/mL (3496.7–7430) vs. 3846.65 ng/mL (2630–4730); *p* = 0.032] (Fig. 1).

The multivariable linear regression analysis showed an independent association with SUVmax for BMI (β = 0.006, 95% CI 0.000–0.0013, *p* = 0.041), the degree of carotid stenosis (β = 0.041, 95% CI 0.000–0.082, *p* = 0.047), and much strongly for sLRP1 levels (β = 0.159, 95% CI 0.062–0.257, *p* = 0.002) (Table 2). In addition, the multivariable logistic regression analysis models showed that, after adjusting for BMI and degree of carotid stenosis, only circulating sLRP1 levels predicted highly inflamed carotid plaques (SUVmax ≥ 2.85 g/mL) (OR = 1.31, 95% CI 1.00–1.01, *p* = 0.046) (Additional file 1: Table S3).

**Table 2:** Multivariable linear regression analysis of the predictors of carotid plaque inflammation (SUVmax)

	*β-coefficient*	95% CI	*β-standardized*	p
logLRP1	0.1592263	0.0618788–0.2565738	0.3989622	0.002
BMI	0.0064457	0.0002674–0.012624	0.2557793	0.041
Stenosis < or ≥ 50%	0.0413429	0.0005251–0.0821606	0.2405786	0.047

*LogLRP1* logarithm of sLRP1, *BMI* body mass index

**Fig. 2:**
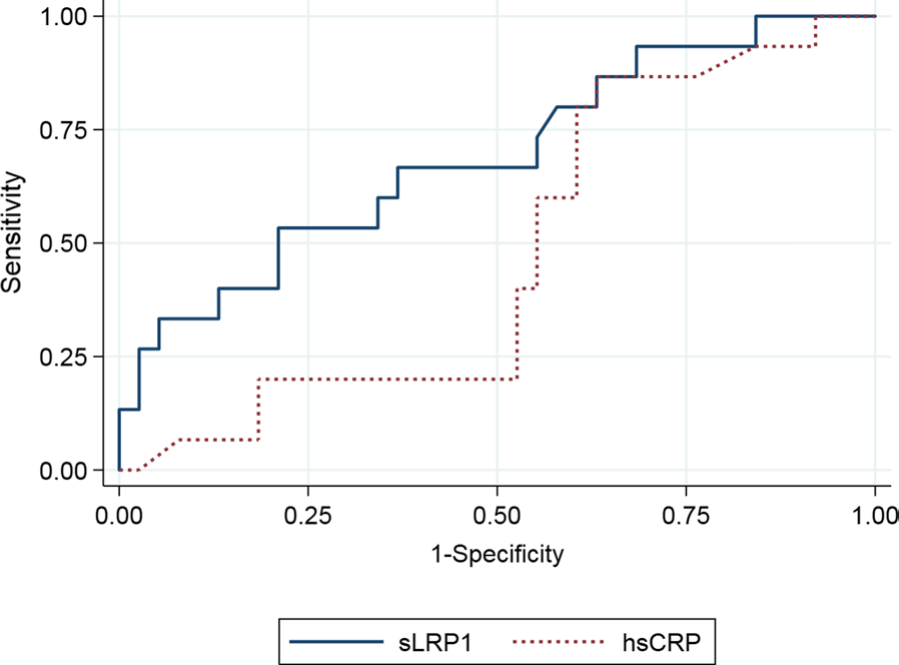
ROC curve analyses of sLRP1 and hsCRP for discrimination of SUVmax > 2.85 g/mL. hsCRP, high sensitivity C reactive protein; sLRP1, soluble low-density lipoprotein receptor-related protein 1

A receiver-operator curve (ROC) analysis was performed to evaluate the sensitivity and specificity of sLRP1 for predicting the presence of highly inflamed carotid plaques (SUVmax ≥ 2.85 g/mL). This analysis showed that the optimal sLRP1 cut-off point was 3500 ng/mL. This sLRP1 value predicted the probability for the presence of highly inflamed plaques with a sensitivity of 80% and a specificity of 42.11%. Compared to hsCRP [area under the curve (AUC) = 0.69 vs. 0.48, *p* = 0.093], the presence of sLRP1 presented a much higher predictive value for highly inflamed plaques (Fig. 2).

### sLRP1 was associated with stroke recurrence

At follow-up after a median of 10 (4–14) months, a total of 9 stroke recurrences were recorded (with stroke occurring in 5 participants before the PET examination, and in 4, after). While the sLRP1 levels in participants with stroke recurrence was higher, this difference was not significant [4430 ng/mL (2630–7430) vs. 3830 ng/mL (2840–4730), *p* = 0.308]. Of note, when only participants with stroke recurrence post-PET during the follow-up were considered, sLRP1 levels were significantly higher at baseline in participants with stroke recurrence than in those without recurrence [6447 ng/mL (4897–11,163) vs. 3713 ng/mL (2793–4730); *p* = 0.018]. In a Cox regression analysis adjusted for carotid artery stenosis (≥ 70% vs. < 70%), the association between sLRP1 levels and post-PET recurrence remained significant (*p* = 0.046).

Other variables that were associated with post-PET recurrence were female sex (*p* = 0.022), having Diabetes Mellitus (*p* = 0.016), and having SUVmax (*p* = 0.008). Unfortunately, the low rate of outcomes prevented us from conducting an adjusted multivariable Cox regression analysis; thus, our results should be interpreted cautiously.

## Discussion

The present study suggests that sLRP1 is a surrogate marker of carotid plaque inflammation. Our main findings are that sLRP1 concentrations i) are independently associated with the degree of plaque inflammation measured by ^18^F-FGD PET, and ii) predict highly inflamed plaques with high sensitivity.

These results are novel and highly relevant because, although previous studies had already shown that sLRP1 circulating levels are associated with the presence of carotid plaque stenosis in experimental models of hypercholesterolemia [22] and in patients with hypercholesterolemia, [15] this is the first time that sLRP1 concentration has been directly and positively associated with carotid plaque inflammation assessed by a highly sensitive method, such as ^18^F-FDG PET. In the previous study conducted with patients with hypercholesterolemia, circulating sLRP1 levels were found to decrease after statin treatment and to increase after statin withdrawal [15]. As all the participants of the current study were intensively treated with statins, it would be expected that sLRP1 levels, as well as their association with inflammation, would be much higher in the absence of statin treatment [29].

Supporting the relationship between sLRP1 concentrations and inflamed carotid plaques, we found a positive correlation between sLRP1 and sICAM-1 levels (with near-significance for sVCAM-1 and FKN). sICAM-1, sVCAM-1, and FKN have been previously associated with carotid plaque inflammation; in particular, sICAM-1 was shown to be the best candidate between these three to predict highly inflamed carotid plaques and the recurrence of stroke within 1 year [10]. Interestingly, the combination of sLRP1 levels (≥ 3500 ng/mL) with sICAM-1 (≥ 240 pg/mL) significantly increased the predictive value for highly inflamed plaques as compared to sLRP1 alone (data not shown). Of note, sLRP1 was closely associated with intraplaque SUVmax but not with stenosis degree, despite the tight association between SUVmax and the degree of stenosis in this group of patients. These results suggest that plaque inflammation is not only linked to stenosis but also that sLRP1 is likely a better indicator of the composition and features of plaque vulnerability than of the plaque size. Along this line, several studies have previously suggested that sLRP1 may be a biomarker that reflects the extra- and intracellular composition of LDL-cholesteryl esters in the atherosclerotic plaques [30–32]. In addition, in previous studies performed with patients with hypercholesterolemia or type 1 diabetes, or the general population, there was a strong correlation between circulating sLRP1 and LDL cholesterol concentrations [11, 15, 33, 34]. We did not observe this correlation in this group of patients, likely due to the treatment of the participants in the study with statins, which efficiently reduces circulating sLRP1 levels [15]. These results do not discard the presence of vascular modified forms of LDL and foam cells in association with plaque inflammation. Indeed, foam cells generated by modified LDL uptake have causal effects on carotid and coronary plaque inflammation in experimental models [14, 22, 23] and in humans [35]. Moreover, foam cells in the human atherosclerotic plaques, which are derived mainly from smooth muscle cells (SMC) and macrophages [36, 37], take up ^18^F-FDG and contribute to the SUVmax measured by PET [38, 39]. All these studies suggest that sLRP1 levels in the participants of the present study are likely associated with a high number of proinflammatory vascular foam cells that avidly take up ^18^F-FDG.

As previously described in the same group of patients [10], results from the present study show that SUVmax is associated with BMI, waist circumference and PACE, in line with the low-grade chronic inflammation ascribed not only to obese, but also to overweight patients [40]. LRP1 levels are also positively correlated with triglycerides and BMI in agreement with the close association between sLRP1 and epicardial fat extension in type 1 diabetes and general population previously reported [33, 34].

Of note, the ROC analysis showed that sLRP1 levels higher than 3,500 ng/mL predicted the presence of highly inflamed carotid plaques (SUVmax ≥ 2.85 g/mL) with high sensitivity. The cut-off points of sLRP1 (3500 ng/mL) found in the present study is similar to sLRP1 values 3210 ng/mL [2690–8310] that were significantly associated with higher levels of BMI, glucose, or triglycerides, a higher REGICOR risk, and an increased incidence of coronary events in our previous case-cohort study [11].

We also observed an association between sLRP1 levels and the risk of stroke recurrence during the (10-month) follow-up. This association was greater and statistically significant when considering only post-PET recurrences. In our cohort, PET/CT was performed within 15 days from inclusion; thus, indirectly, we may assume that sLRP1 levels were associated with latter events rather than early recurrences related to an unstable ruptured carotid plaque. Unfortunately, the low rate of outcomes prevented us from conducting an adjusted multivariable Cox regression analysis; thus, these results should be interpreted cautiously.

The results of this study, together with a previous study showing that the sLRP1 concentration is independently associated with CAD risk, [11] support the potential of sLRP1 as a dual prognostic biomarker of carotid and coronary artery events. A recent meta-analysis comparing the relationship between phenotypic manifestation of coronary and carotid atherosclerosis suggests that knowing the state of carotid arteries whenever CAD is suspected has clinical utility [41, 42]. Based on these results, further studies in large cohorts are warranted to determine this putative predictive role of sLRP1 as a dual biomarker of carotid and coronary atherosclerosis.

The strengths of our study include the strict carotid vascular characterization through ^18^F-FDG PET, the prospective follow-up of patients, and the control for potential covariates.

### Limitations

Limitations include the limited number of participants in the study due to narrow eligibility criteria, as well as the limited number of recurrences due to the relatively short follow-up. Further studies with increased numbers of participants and long-term follow-up are required to validate the potential sLRP1 diagnostic and prognostic value in the context of carotid atherosclerosis and stroke.

## Conclusions

**Fig. 3:**
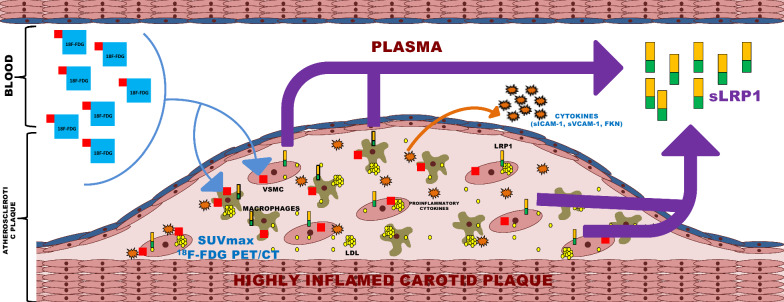
The progression of atherosclerotic plaque is associated with an increased number of active cells in the arterial intima (macrophages and smooth muscle cells) that avidly take up ^18^F-FDG (SUVmax). Many of these cells become foam cells by taking up atherogenic lipoproteins retained in the intimal extracellular matrix, acquiring a highly proinflammatory phenotype that release cytokine and sLRP1 to the circulation. The results of the present study show the close relationship between sLRP1 and proinflammatory cytokines (sICAM-1, sVCAM-1 and FKN) in the blood, as well as between circulating sLRP1 levels and intra-plaque inflammation measured by PET in patients with a recent ischemic stroke. ^*18*^*F-FDG* 18F-fluorodeoxyglucose, *SUVmax* standardized uptake value, *sLRP1* soluble low-density lipoprotein receptor-related protein 1, *sICAM-1* soluble intercellular cell adhesion molecule 1, *sVCAM-1* soluble vascular adhesion molecule 1 (sVCAM-1), *FKN* fractalkine

In summary (Fig. 3), this study provides new evidence that plasma sLRP1 is independently associated with carotid plaque inflammation and with the probability of presenting SUVmax ≥ 2.85 g/mL. In addition, here, we show that sLRP1 levels are higher in patients with stroke recurrence during follow-up. Together, these results support the role of sLRP1 in inflammation and vascular pathology by revealing that sLRP1 could be a surrogate biomarker of carotid plaque inflammation in patients with recent ischemic stroke. This knowledge paves the way for further research addressing the putative use of sLRP1, alone or in combination with other plasma biomarkers, for selecting patients eligible for ^18^F-FDG PET. This biomarker could also be clinically useful for predicting the risk of ischemic stroke and other cardiovascular diseases as a complementary information to ^18^F-FDG PET.

## Supplementary Information

**Additional file 1: Table S1.** Clinical characteristics and biochemical parameters of patients dichotomized according to carotid artery stenosis. BMI: Body mass index; HDL: High density lipoproteins; hsCRP: high-sensitivity C-reactive protein; LDL: Low density lipoproteins; NIHSS: National institute of Health Stroke Scale; PACE: Physician-based Assessment and Counseling for Exercise; PREDIMED: PREvención con DIeta MEDiterránea; SUVmax: Maximum standarized Uptake Value; TC: Total cholesterol; TG: Triglycerides. A chi2 test was used to determine the frequencies and the p value of categorical variables and a Mann-Whitney test to determine the p value for quantitative variables. **Table S2.** Bivariate linear regression analyses of the association between clinical variables and carotid inflammation (SUVmax). BMI: Body mass index; CRP: C reactive protein; HDL: High density lipoproteins; LDL: Low density lipoproteins; LogLRP1: logarithm of sLRP1; NIHSS: National institute of Health Stroke Scale; PACE: Physician-based Assessment and Counseling for Exercise; PREDIMED: PREvención con DIeta MEDiterránea; sLRP1: Soluble low-density lipoprotein receptor-related protein 1; TC: Total cholesterol; TG: Triglycerides. A linear regression was used to determine the b-coefficient, the β- standardized and the p value. **Table S3.** Logistic regression analyses of the association between sLRP1 and metabolic variables with carotid inflammation in patients with SUVmax ≥ 2.85 g/ml. sLRP1: Soluble LRP1; Stenosis < or = > 50%: patients dichotomized as having 50% or more stenosis or less than 50% stenosis; BMI: Body mass index. A logistic regression was used to determine the odds-ratio and the p value.

## Data Availability

The datasets used and/or analyzed during the current study are available from the corresponding author on reasonable request.

## Abbreviations

BMI: Body mass index
CAD: Coronary artery disease
EDTA: Ethylenediaminetetraacetic acid
^18^F-FDG: ^18^F-fluorodeoxyglucose
FKN: Fractalkine
Hba1c: Glycated hemoglobin
HDLc: High-density lipoprotein cholesterol
hsCRP: High-sensitive C reactive protein
ICA: Internal carotid artery
LDLc: Low-density lipoprotein cholesterol
mRS: Modified Rankin scale
NIHSS: National Institutes of Health Stroke Scale
PACE: Physician-based assessment and counseling for exercise
PET: Positron emission tomography
REGICOR: Registre Gironi del Cor
sICAM-1: Soluble intercellular adhesion molecule-1
sLRP1: Soluble low-density lipoprotein receptor-related protein 1
SMC: Smooth muscle cells
SUVmax: Maximal standardized uptake value
sVCAM-1: Soluble vascular cell adhesion molecule 1
TC: Total cholesterol
TG: Triglycerides
TIA: Transient ischemic attack
tPA: Tissue plasminogen activator
