# Supplementary material for: Soluble low-density lipoprotein receptor-related protein 1 as a surrogate marker of carotid plaque inflammation assessed by 18F-FDG PET in patients with a recent ischemic stroke
Source: J Transl Med. 2023 Feb 19;21:131. doi: 10.1186/s12967-022-03867-w (PMC9940334; doi:10.1186/s12967-022-03867-w)
Supplement: Supplementary file 1 — Additional file 1: Table S1. Clinical characteristics and biochemical parameters of patients dichotomized according to carotid artery stenosis. BMI: Body mass index; HDL: High density lipoproteins; hsCRP: high-sensitivity C-reactive protein; LDL: Low density lipoproteins; NIHSS: National institute of Health Stroke Scale; PACE: Physician-based Assessment and Counseling for Exercise; PREDIMED: PREvención con DIeta MEDiterránea; SUVmax: Maximum standarized Uptake Value; TC: Total cholesterol; TG: Triglycerides. A chi2 test was used to determine the frequencies and the p value of categorical variables and a Mann-Whitney test to determine the p value for quantitative variables. Table S2. Bivariate linear regression analyses of the association between clinical variables and carotid inflammation (SUVmax). BMI: Body mass index; CRP: C reactive protein; HDL: High density lipoproteins; LDL: Low density lipoproteins; LogLRP1: logarithm of sLRP1; NIHSS: National institute of Health Stroke Scale; PACE: Physician-based Assessment and Counseling for Exercise; PREDIMED: PREvención con DIeta MEDiterránea; sLRP1: Soluble low-density lipoprotein receptor-related protein 1; TC: Total cholesterol; TG: Triglycerides. A linear regression was used to determine the b-coefficient, the β- standardized and the p value. Table S3. Logistic regression analyses of the association between sLRP1 and metabolic variables with carotid inflammation in patients with SUVmax ≥ 2.85 g/ml. sLRP1: Soluble LRP1; Stenosis < or = > 50%: patients dichotomized as having 50% or more stenosis or less than 50% stenosis; BMI: Body mass index. A logistic regression was used to determine the odds-ratio and the p value. [file 12967_2022_3867_MOESM1_ESM.docx]

**Additional file tables**

**Table S1. Clinical characteristics and biochemical parameters of patients dichotomized according to carotid artery stenosis.** BMI: Body mass index; HDL: High density lipoproteins; hsCRP: high-sensitivity C-reactive protein; LDL: Low density lipoproteins; NIHSS: National institute of Health Stroke Scale; PACE: Physician-based Assessment and Counseling for Exercise; PREDIMED: PREvención con DIeta MEDiterránea; SUVmax: Maximum standarized Uptake Value; TC: Total cholesterol; TG: Triglycerides. A chi2 test was used to determine the frequencies and the p value of categorical variables and a Mann-Whitney test to determine the p value for quantitative variables.

|  | Total | < 50% stenosis  (n = 27) | ≥ 50% stenosis  (n = 37) | p |
| --- | --- | --- | --- | --- |
| Hypertension, n (%) | 53 | 26 (96.30) | 27 (72.97) | **0.015** |
| Diabetes Mellitus, n (%) | 27 | 9 (33.30) | 18 (48.70) | 0.220 |
| Hyperlipidemia, n (%) | 43 | 20 (74.10) | 23 (62.20) | 0.316 |
| Prior ischemic heart disease, n (%) | 17 | 7 (25.90) | 10 (27.00) | 0.922 |
| Prior stroke, n (%) | 11 | 5 (18.50) | 6 (16.20) | 0.809 |
| Prior peripheral vascular disease, n (%) | 12 | 5 (18.50) | 7 (18.90) | 0.968 |
| Waist Circumference (cm), md (IQR) | 98 (89-102) | 101 (90-105.5) | 98 (87.5-101.5) | 0.294 |
| PREDIMED score, md (IQR) | 9 (6-10) | 9 (5-10) | 9 (7-9) | 0.609 |
| PACE, md (IQR) | 3 (2-4.5) | 3 (2-4) | 2 (1.5-5) | 0.746 |
| NIHSS, md (IQR) | 2 (0.0-4.6) | 3 (1-6) | 2 (0-3) | 0.324 |
| SUVmax, md (IQR) | 2.62 (2.3-3.08) | 2.39 (2.23-2.69) | 2.74 (2.41-3.23) | **0.034** |
| BMI, md (IQR) | 25.82 (23.53-27.66) | 25.92 (23.7-27.72) | 25.75 (21.97-27.66) | 0.659 |
| TC, md (IQR) | 148.61 (122.63-179.18) | 145 (124.23-189.63) | 153.64 (121.02-177.63) | 0.957 |
| LDL, md (IQR) | 83.58 (57.5-113.92) | 86.55 (58.00-121.80) | 76.81 (56.70-108.85) | 0.708 |
| HDL, md (IQR) | 39.86 (31-50.65) | 39.47 (31-50.26) | 40.25 (31.00-51.04) | 0.838 |
| TG, md (IQR) | 102.78 (80.61-102.76) | 97 (77-134) | 105 (84-141.6) | 0.559 |
| hsCRP (mg/dl), md (IQR) | 4.95 (2.2-11.55) | 3.6 (1.9-7.1) | 6.2 (2.9-19.1) | **0.047** |
| Prior antiplatelet therapy, n (%) | 37 | 15 (55.60) | 22 (59.50) | 0.755 |
| Prior oral anticoagulant, n (%) | 1 | 0 (0.0) | 1 (2.70) | 0.389 |
| Prior statins, n (%) | 35 | 15 (55.6) | 20 (54.1) | 0.905 |

**Table S2. Bivariate linear regression analyses of the association between clinical variables and carotid inflammation (SUVmax).** BMI: Body mass index; CRP: C reactive protein; HDL: High density lipoproteins; LDL: Low density lipoproteins; LogLRP1: logarithm of sLRP1; NIHSS: National institute of Health Stroke Scale; PACE: Physician-based Assessment and Counseling for Exercise; PREDIMED: PREvención con DIeta MEDiterránea; sLRP1: Soluble low-density lipoprotein receptor-related protein 1; TC: Total cholesterol; TG: Triglycerides. A linear regression was used to determine the b-coefficient, the β- standardized and the p value.

| Variable | *β-coefficient* | 95% CI | *β-standardized* | p |
| --- | --- | --- | --- | --- |
| Bivariate analyses |  |  |  |  |
| Age | 0.0011171 | -0.0013735-0.0036078 | 0.13 | 0.372 |
| Sex | 0.0363926 | -0.0171825- 0.0899677 | 0.19 | 0.179 |
| **BMI** | 0.0082856 | 0.0015953- 0.014976 | 0.33 | **0.016** |
| **Waist Circumference** | 0.0035351 | 0.001165- 0.0059053 | 0.42 | **0.004** |
| **Current smoking** | -0.0691765 | -0.1215743 -0.0167787 | -0.35 | **0.011** |
|  |  |  |  |  |
|  |  |  |  |  |
| **PACE** | -0.0149329 | -0.0277212 -0.0021445 | -0.31 | **0.023** |
| PACE 4_8 | -0.0433445 | -0.0899492 -0.0032602 |  | 0.068 |
| PREDIMED | -0.0050621 | -0.0157028- 0.0055786 | -0.13 | 0.344 |
| Hypertension | 0.042409 | -0.0276078- 0.1124259 | 0.17 | 0.230 |
| Diabetes Mellitus | 0.0245255 | -0.0235012- 0.0725521 | 0.14 | 0.310 |
| Hyperlipidemia | 0.0191346 | -0.0329698 -0.0712389 | 0.10 | 0.464 |
| Prior ischemic heart disease | -0.0297034 | -0.0836036- 0.0241968 | -0.15 | 0.274 |
| Prior ischemic stroke | -0.0300166 | -0.0935038- 0.0334707 | -0.13 | 0.347 |
| Prior peripheral vascular disease | 0.0089574 | -0.0524524- 0.0703672 | 0.04 | 0.771 |
| Prior antiplatelet therapy | -0.0203897 | -0.0688547- 0.0280753 | -0.12 | 0.402 |
| Prior oral anticoagulants | -0.0707558 | -0.246373- 0.1048615 | -0.11 | 0.422 |
| Prior statins | 0.0003076 | -0.0484951- 0.0491103 | 0.00 | 0.990 |
| Stenosis < or = > 50% | 0.0407319 | -0.0062009- 0.0876647 | 0.24 | 0.087 |
| NIHSS | -0.0039828 | -0.0093857- 0.00142 | -0.20 | 0.145 |
| **logLRP1** | 0.1921105 | 0.0937693- 0.2904517 | 0.48 | **<0.001** |
| TC | 0.0001912 | -0.0003547- 0.000737 | 0.10 | 0.485 |
| TG | 0.0003841 | -0.0001653- 0.0009334 | 0.20 | 0.166 |
| LDL | 0.000077 | -0.0006184- 0.0007723 | 0.03 | 0.825 |
| HDL | 0.0006344 | -0.0011658- 0.0024347 | 0.11 | 0.481 |
| CRP | 0.0003736 | -0.0023259- 0.0030731 | 0.04 | 0.781 |

**TABLE S3. Logistic regression analyses of the association between sLRP1 and metabolic variables with carotid inflammation in patients with SUsVmax ≥ 2.85 g/ml.** sLRP1: Soluble LRP1; Stenosis < or = > 50%: patients dichotomized as having 50% or more stenosis or less than 50% stenosis; BMI: Body mass index. A logistic regression was used to determine the odds-ratio and the p value.

| Variable | *Odds-ratio* | 95% CI | p |
| --- | --- | --- | --- |
| LogLRP1 | 1.31 | 1.00 - 1.01 | 0.046 |
| BMI | 1.19 | 0.94 - 1.41 | 0.176 |
| Stenosis < or = > 50% | 4.08 | 0.91 - 17.72 | 0.067 |
